# Malposition of septum primum in isolated dextrocardia: unique and rare form of anomalous pulmonary venous return in association with partial absence of pericardium-case report

**DOI:** 10.1186/s13019-021-01591-y

**Published:** 2021-07-31

**Authors:** Neerod Kumar Jha, Haitham Talo, Laszlo Kiraly, Nishant Shah, Aref Al Hakami, Zafar Althaf Azeez, Ajitha Kumari Kamala Bai

**Affiliations:** 1 Division of Pediatric Cardiac Surgery, Sheikh Khalifa Medical City, PO BOX 51900, Abu Dhabi, United Arab Emirates; 2 Pediatric Cardiology, Sheikh Khalifa Medical City, Abu Dhabi, United Arab Emirates

**Keywords:** Pulmonary, Cardiac, Vein, Dextrocardia, Pericardium, Surgery, Return, Absent, Anomalous, Embryology

## Abstract

**Background:**

Total anomalous pulmonary venous return (TAPVR) refers to an anomaly in which all of the pulmonary veins drain directly or indirectly to the systemic venous circulation. However, unusual types constitute approximately 5% or less of TAPVRs and there may be obstruction or discontinuity of pulmonary vein at various levels.

**Case presentation:**

A 3-month-old infant was presented to us with history of poor feeding, respiratory distress and desaturations. The routine echocardiographic investigation initially confirmed the diagnosis of an atrial septal defect with dextrocardia. However, due to disproportionate severity of symptoms and congestive heart failure a cardiac computer tomography angiogram was done that revealed a rare finding of connection of pulmonary veins fused with the posterior atrium, but on the rightward side of the deviated atrial septum. Therefore, pulmonary veins entered a sinus that drains directly into the right atrial superior-posterior wall. During surgical repair, we found an area of absent pericardium in the diaphragmatic surface of the heart. The patient underwent total repair of the TAPVR and patch reconstruction of the pericardial defect. The patient is doing well at 6-month follow up.

**Conclusions:**

The septum primum malposition defect resulting in TAPVR is a very rare congenital anomaly that can be rarely seen without any heterotaxy. The anomalous features including absent pericardium and dextrocardia were present in this patient have not been described previously with TAPVR. Therefore, we have hypothesized the embryological correlation of absent pericardium and cardiac malposition in such case. Transthoracic echocardiography with Doppler interrogation is a reliable method for diagnosing this condition. In case of suboptimal echocardiographic image due to cardiac position, unclear anatomy or unexplained symptoms, advanced imaging such as computer tomographic angiography or cardiac magnetic resonance imaging can be very helpful. Preoperative proper diagnosis of this anomaly facilitates successful surgical management with excellent outcome.

## Background

Total anomalous pulmonary venous return (TAPVR) refers to an anomaly in which all of the pulmonary veins drain directly or indirectly to the systemic venous circulation [1, 2]. There are various types of such defects described in the literature [1]. However, unusual types constitute approximately 5% or less of TAPVRs and there may be obstruction at various levels or discontinuity of pulmonary veins.

The intra-cardiac type constitutes 25% of all TAPVRs and mostly drains into the coronary sinus [2]. The present patient had extremely rare finding of connection of pulmonary veins fused with the posterior atrium, but on the rightward side of the deviated atrial septum. Therefore, pulmonary veins entered a sinus that drains directly into the right atrial superior-posterior wall. In addition, there was dextrocardia and absent pericardium on the diaphragmatic surface of the heart. Similar findings have not been described previously and therefore, we have hypothesized the embryological correlation of absent pericardium in such case.

## Case presentation

A 3-month-old infant was presented with history of poor feeding, respiratory distress and congestive heart failure. There was no remarkable past medical or family history. On examination, the vital parameters were normal. Excepting far, the peripheral oxygen saturation was around 85–90% on room air.

**Fig. 1:**
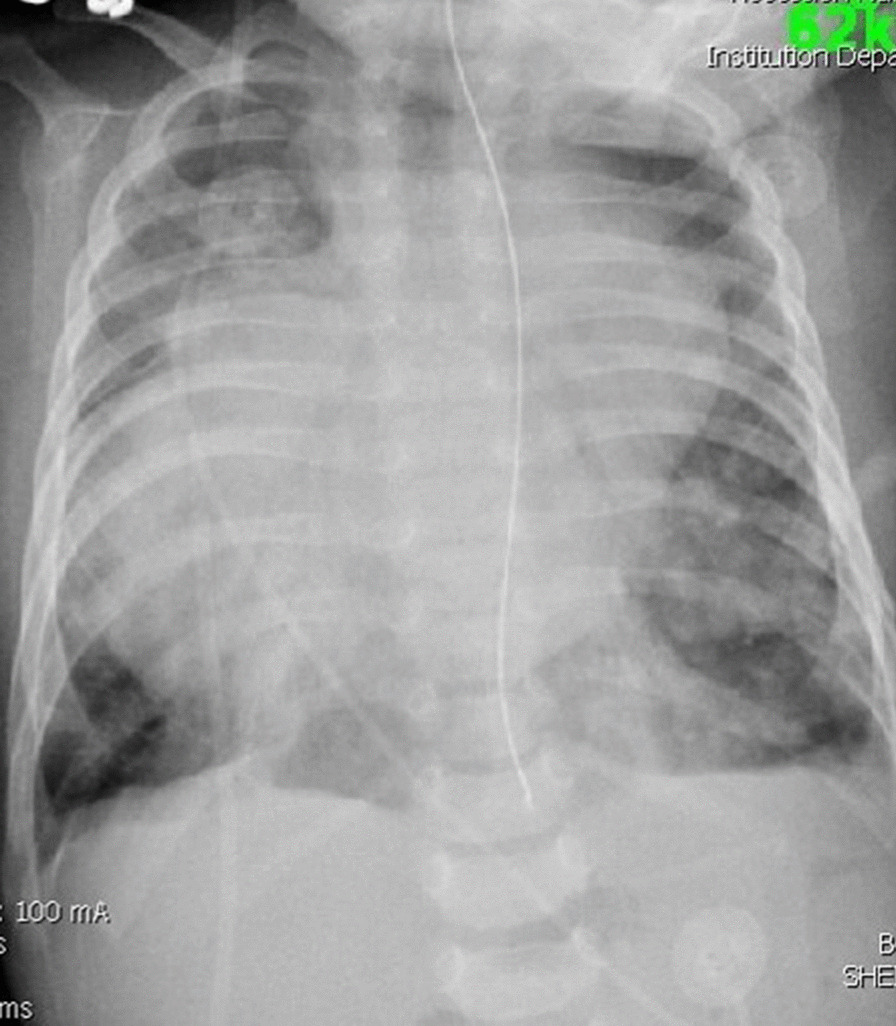
Plain chest radiograph showing features of cardiomegaly, dextrocardia and lung congestion

A chest X ray revealed double contoured heart with soft tissue shadow in the mediastinum suggestive of atrial enlargement and cardiomegaly. The lung fields were congested. The apex of the heart was on the right of the midline (Fig. 1).

A transthoracic echocardiography showed dextrocardia, situs solitus, normal great arteries, large secundum atrial septal defect (ASD) with bi-directional shunt, mild tricuspid regurgitation, pulmonary hypertension, dilated right-sided cardiac chambers, ventricular septal deviation to the left, normal biventricular function and right-caval axis. Congestive heart failure and pulmonary hypertension was unusual for the initial findings of an ASD and therefore advanced cardiac imaging was planned to further delineate the morphology and to set up management plan.

**Fig. 2:**
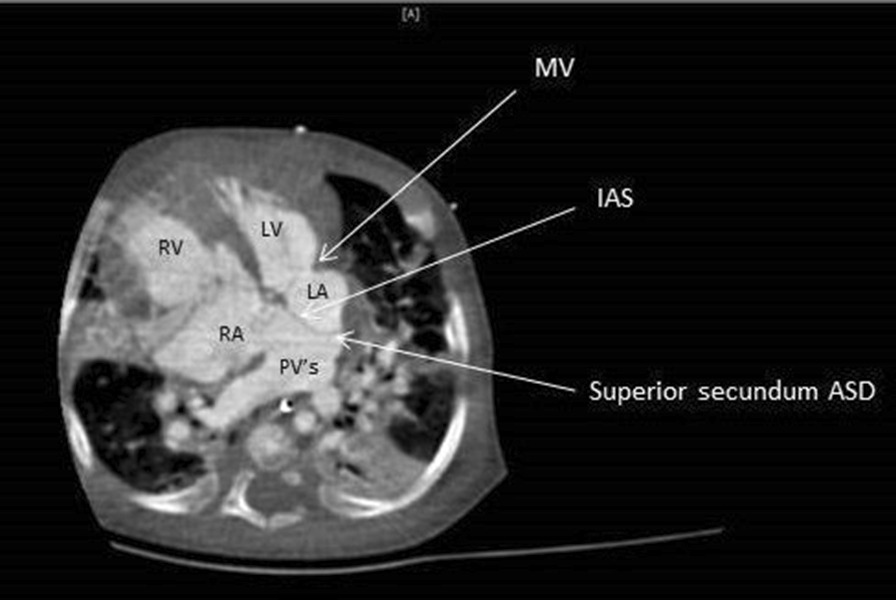
CT angiogram showing TAPVR (MV—mitral valve, IAS—inter atrial septum, ASD—atrial septal defect, PV—pulmonary veins opening in to the RA, RV—right ventricle, LV—left ventricle, RA—right atrium, LA—left atrium)

**Fig. 3:**
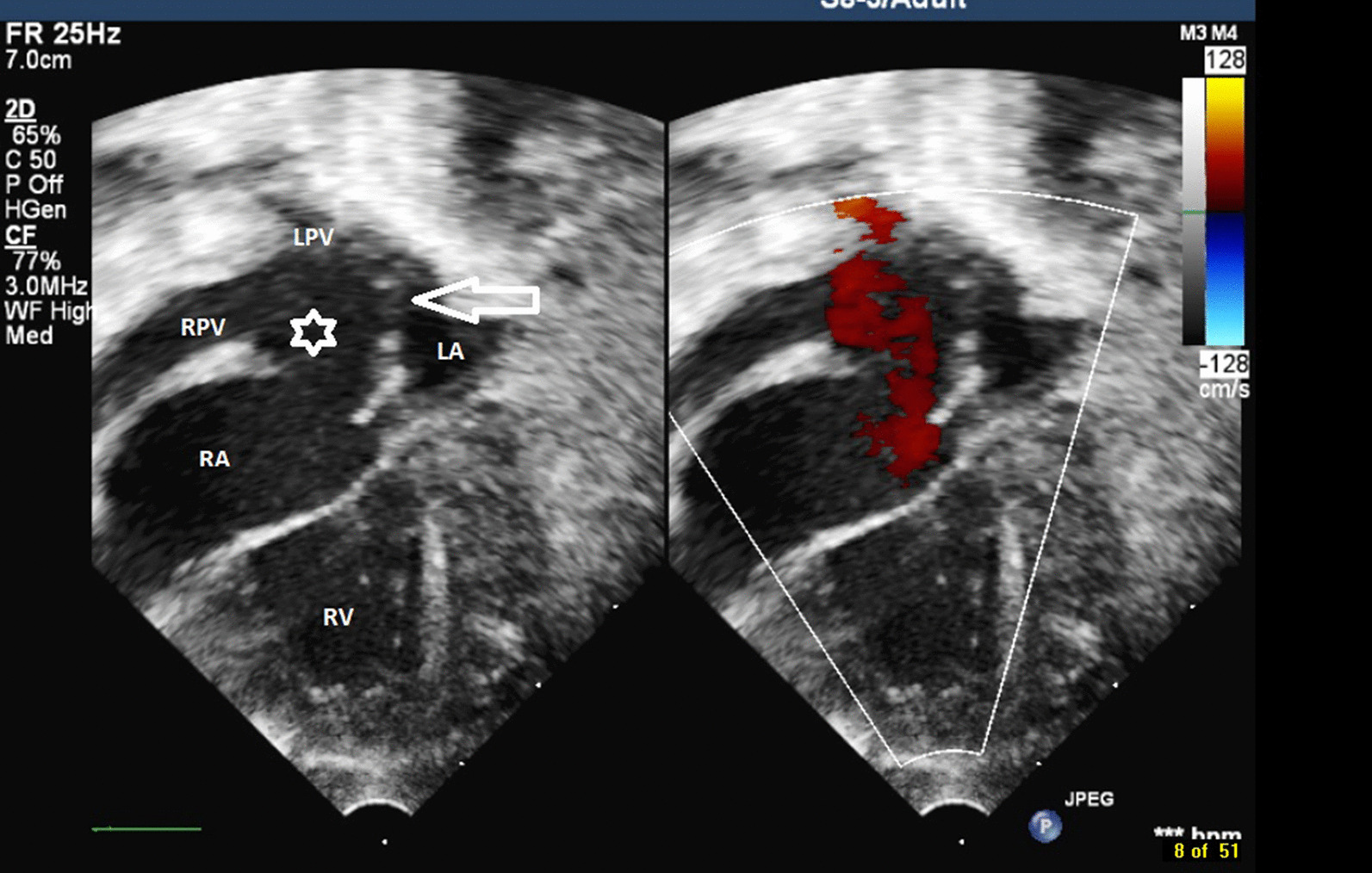
Preoperative 2-D echocardiogram including colour Doppler showing TAPVR into the RA (RPV—right pulmonary veins, LPV—left pulmonary veins, RA—right atrium, LA—left atrium, RV—right ventricle, white star—TAPVR to the RA, White arrow—inter atrial communication)

The cardiac computer tomographic (CT) angiogram showed significant deviation of atrial primum septum to the left. The dilated pulmonary veins from either sides entered and drained into the right-sided atrium through a sinus on the right side of the left-ward deviated interatrial septum. (Fig. 2). The similar findings then were re-confirmed on a repeated echocardiogram (Fig. 3). The coronary sinus and mitral valve were normal.

An urgent surgical repair was performed via median sternotomy using cardiopulmonary bypass, cardioplegic arrest and systemic hypothermia (32 °C). Intracardiac repair included, resection of adjacent wall of the common pulmonary venous sinus into the atrium. An atrial separation patch of autologous pericardium baffled the pulmonary venous flow to the left atrium and also closed the ASD. Incidentally, we found an area of the absent pericardium on the right side of the chest, below the apex of the ventricle extending towards the tendinous center of the diaphragm. An autologous pericardial patch was used to repair the defect which was 5 × 3 cm in size.

**Fig. 4:**
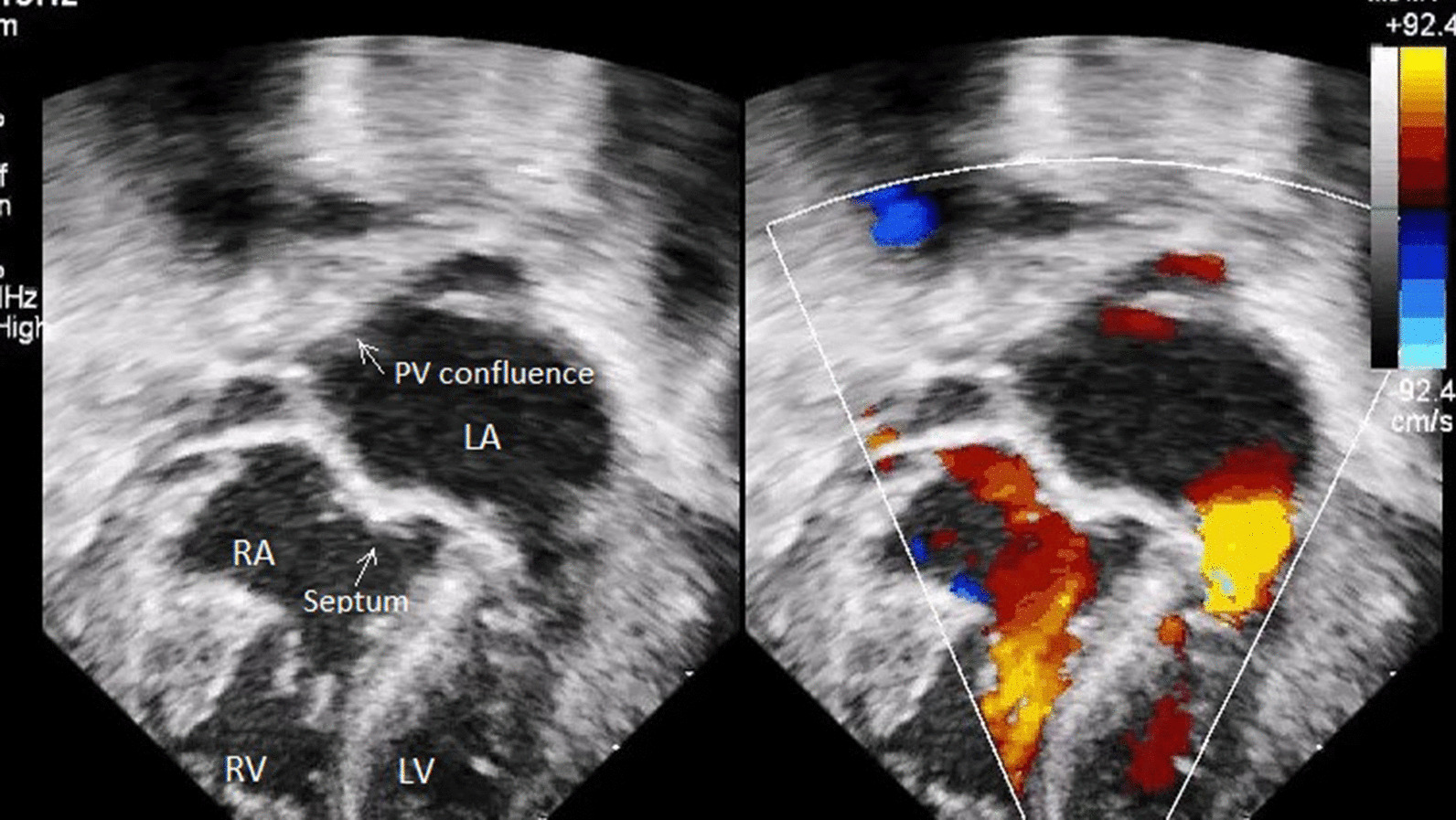
Postoperative 2-D echocardiogram including colour Doppler showing partition of atrium and diversion of pulmonary venous drainage into the left atrium (LA—left atrium, RA—right atrium, RV—right ventricle, LV—left ventricle, PV—pulmonary veins)

**Fig. 5:**
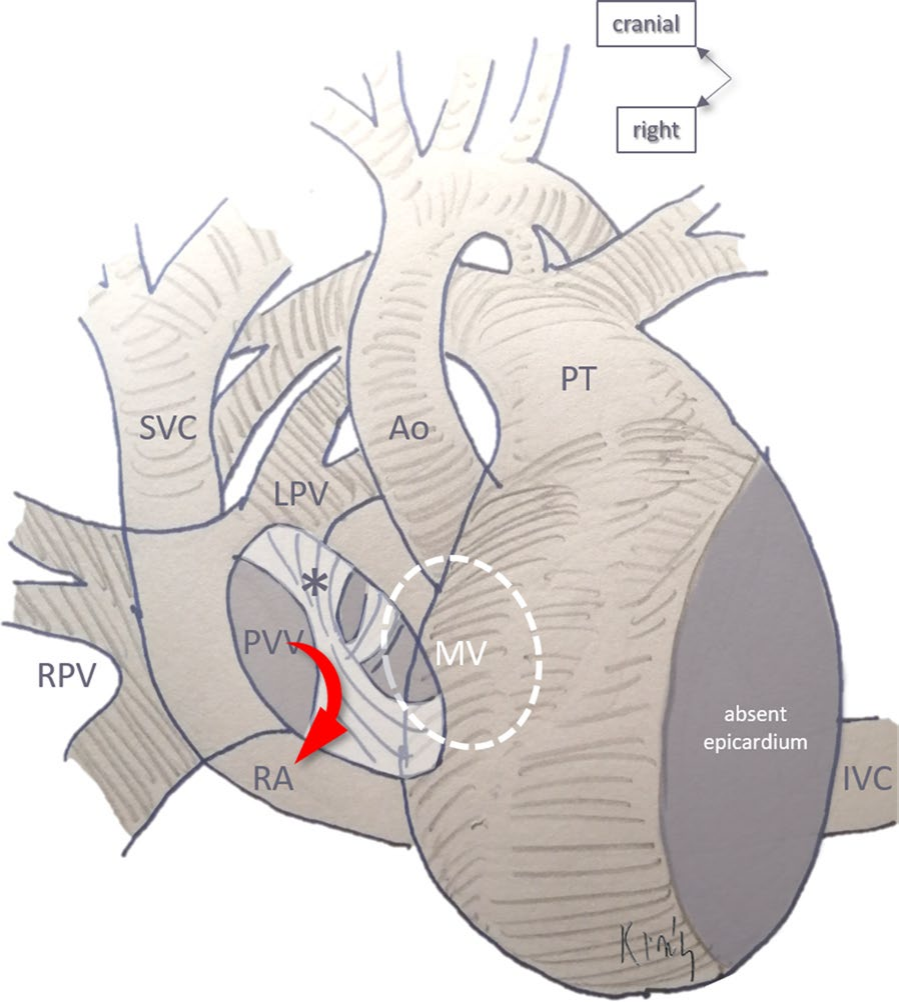
Artist’s impression of the surgical anatomy. The pulmonary venous inflow (PVV) is redirected into the right atrium (RA) by the flap (*) of the fossa ovalis. Abbreviations: *Ao* aorta, *IVC* inferior vena cava, *LPV* left-sided pulmonary veins, *PT* pulmonary trunk, *PVV* pulmonary veins, *RA* right atrium, *SVC* superior vena cava

**Fig. 6:**
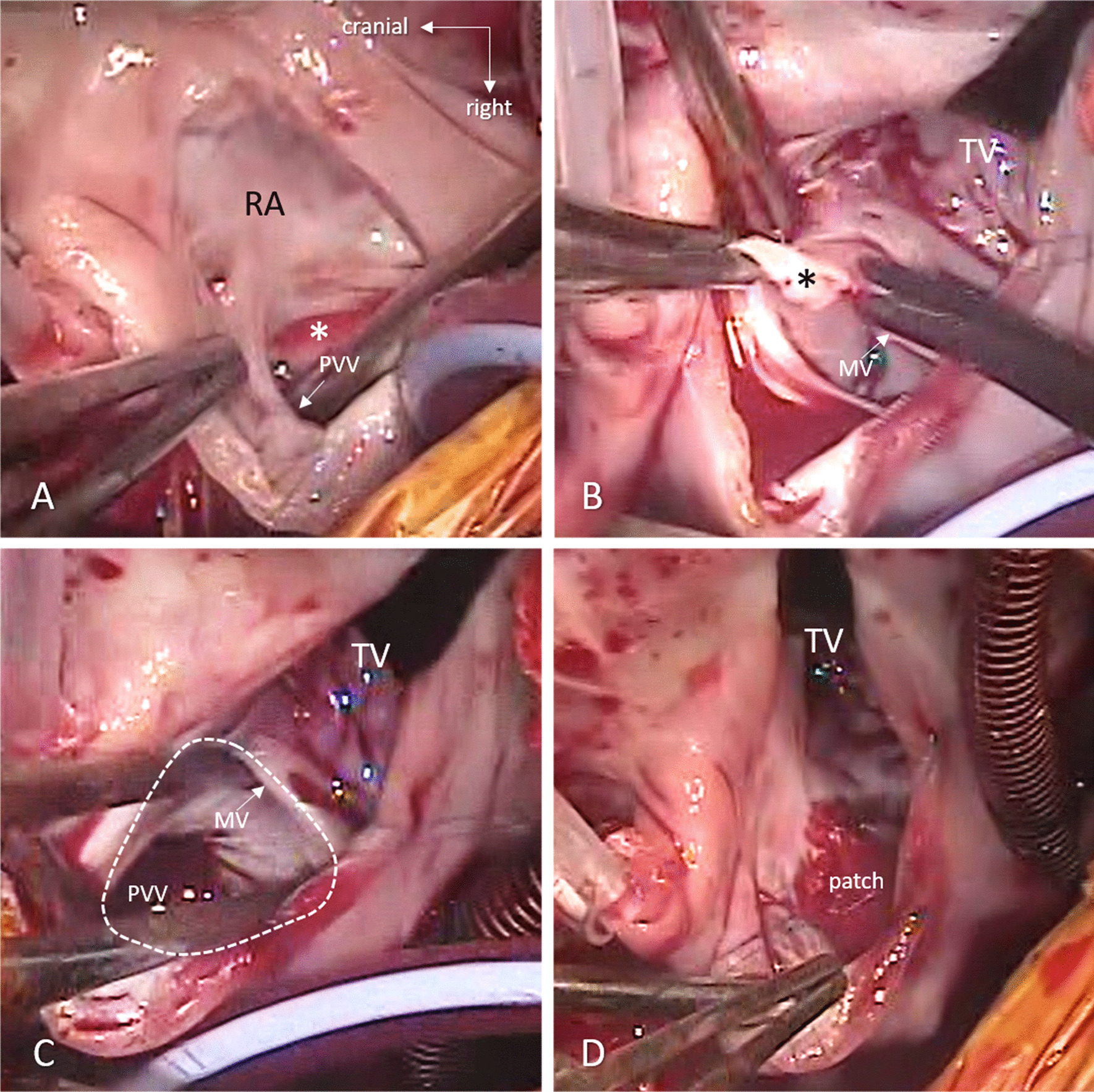
Steps of the intracardiac repair taken from the intraoperative video-recording, from the operator’s view. **A** Cribrous flap of the fossa ovalis (*) separates the inflow of the pulmonary veins (PVV) from the left atrium. **B** Resection of the flap (*) opens up the view towards the mitral valve (MV). **C** Smooth pathway is created from the pulmonary veins (PVV) to the mitral valve (MV). The interatrial communication (dotted line) is closed (**D**) with an autologous pericardium patch

**Fig. 7:**
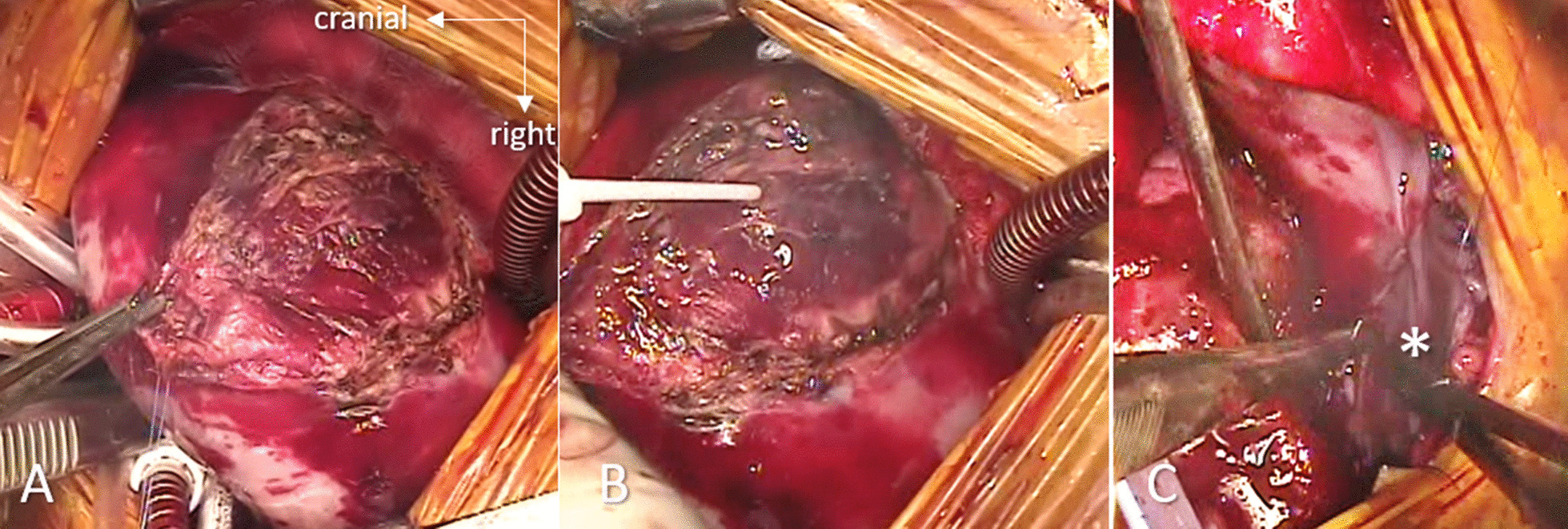
Steps of the extracardiac repair taken from the intraoperative video-recording, from the operator’s view. **A** A large defect of the epicardium is seen revealing the raw myocardium. **B** Epicardium defect is sealed with fibrin glue. **C** Corresponding defect of the diaphragmatic parietal pericardium is repaired with a patch (*) of autologous pericardium

The patient recovered well and was discharged from the hospital on usual cardiac medications, 2 weeks after repair. At 6-month follow up his condition and echocardiographic findings shows symptomatic relief, normal vital parameters and no residual defects in the echocardiography with normal hemodynamic parameters (Fig. 4). The artist’s impression of the surgical anatomy (Fig. 5), operative photographs of various steps of intra-cardiac surgical repair (Fig. 6) and pictures showing stages of extra-cardiac surgical repair including pericardial defect reconstruction (Fig. 7) represent finer details from a surgical perspective.

## Discussion

The primordial lung buds drain through the common cardinal and umbilicovitelline veins. The right cardinal systems ultimately become right superior venacava and the azygos vein. The left cardinal veins ultimately make left superior vena cava and the coronary sinus. The umbilicovitelline veins become inferior vena cava, portal vein and the ductus venosus. The pulmonary veins drains to the left atrium by incorporating in the posterior wall. Any deviations in the septum primum (malposition) may alter the pulmonary venous drainage across the septum and causes an anomalous drainage [1, 2]. Similarly, the embryological malalignment of the primitive systemic and pulmonary venous systems not only alter the relationships of all the structures but may also lead to confluent or non-confluent pulmonary venous connections to the systemic veins [1]. A secondary affect may be seen in the pleuroperitoneal or pleuropericardial membranes and their fusion, leading to partial or complete absence of pericardium or pleural membranes [3]. Hypothetically, the presence of dextrorotation of the ventricular loop could be another reason of malfusion of the pleuropericardial membranes that results in a pericardial defect.

The classic cardiac type of the TAPVR drain into the coronary sinus. However, direct connection of the pulmonary veins to the morphological right atrium is extremely rare in the absence of right atrial isomerism [1, 2]. In our case, there was a malalignment of the atrial septum leading to the TAPVR into the right atrium in association with dextrocardia. In this situation, the blood flow to the left heart chambers is dependent on the size of the interatrial septal communication [4, 5].

The septum primum malposition defect is a very rare congenital anomaly. Absent or faulty development of septum secundum leads to malposition of septum primum that may be displaced toward the anatomic left atrium. This displacement of the upper border of the septum primum will result in the incorporation of some or even all the pulmonary veins into the morphologically right atrium. This leads to normally connected pulmonary veins to back of the atrial wall draining abnormally in the right atrium. The absence of septum secundum results into small inter atrial communication along the posterior and superior wall of left atrium and displaced mobile upper border of the septum primum [5].

Routine echocardiography is usually sufficient to delineate the anatomy and the physiology in most of the patients with simple TAPVRs. However, due to presence of suspicious clinical correlations or suspected unusual or rare nature of this entity there is always a need to explore better spatial resolution and exclude associated anomalies. In our patient, the initial findings on echocardiography was a large ASD with pulmonary hypertension. However, the symptoms and pulmonary hypertension were not consistent with the echocardiographic findings and therefore, advanced cardiac imaging was planned. Cardiac CT angiography or cardiac magnetic resonance imaging (MRI) both modalities can provide necessary information. Authors decided to proceed with CT because of relatively unstable child from significant respiratory distress. The cardiac MR angiography provides better anatomic or physiologic detail and should be used in a stable situation. Both modalities provide necessary information to plan a suitable surgical approach [6].

The surgery should be aimed to redirect the blood to the left atrium, close the inter-atrial communication and correct additional anomalies. The urgent surgical approach is warranted.

Pericardial defects are exceedingly rare with an incidence of less than 1 in 10,000 as per autopsy studies [3]. The diagnosis is mainly incidental due to the fact that majority of the patients are asymptomatic. Unilateral defects are right sided and constitute 17% of all the defects [3]. Our patient had right sided partial absence of pericardium below the right-sided apex of the ventricle and over the diaphragm. A pericardial patch reconstruction of the small defect was done during the surgery. However, it is difficult to explain the benefit of surgery or significance of this finding. On a literature search, we found that the pericardial defects have been found to be associated with anomalous pulmonary venous drainage and sinus venosus atrial septal defects [3]. We may also hypothesize that absence of the pericardium introduced rightward rotation of the ventricular loop, consequently shifted the planes of the developing septa that might have contributed to the anomalous attachment of the pulmonary veins. The patch repair was done to halt any herniation of the thoracic structures.

## Conclusions

The septum primum malposition defect is a very rare congenital anomaly. This abnormality is predominantly seen in patients with visceral heterotaxy, although it can be rarely seen without any heterotaxy. Transthoracic echocardiography with Doppler interrogation is a reliable method for diagnosing this anomaly. In case of suboptimal echocardiographic image due to cardiac position, unclear anatomy, or disproportionate symptoms, advanced imaging such as cardiac CT angiography or cardiac MRI can be very helpful. Preoperative proper diagnosis of this anomaly facilitates successful surgical management with excellent outcome. Associated finding of absent pericardium in presence of cardiac malposition and TAPVR needs further embryological explanations for better understanding.

## Data Availability

The data used to support this publication is available with authors.

## Abbreviations

TAPVR: Total anomalous pulmonary venous return
CT: Computer tomography
ASD: Atrial septal defect
MR: Magnetic resonance
